# Creating a zero-order resonator using an optical surface transformation

**DOI:** 10.1038/srep21333

**Published:** 2016-02-18

**Authors:** Fei Sun, Xiaochen Ge, Sailing He

**Affiliations:** 1 State Key Laboratory of Modern Optical Instrumentations, Centre for Optical and Electromagnetic Research, JORCEP, East Building #5,Zijingang Campus, Zhejiang University, Hangzhou 310058, China; 2 Department of Electromagnetic Engineering, School of Electrical Engineering, Royal Institute of Technology (KTH), S-100 44 Stockholm, Sweden

## Abstract

A novel zero-order resonator has been designed by an optical surface transformation (OST) method. The resonator proposed here has many novel features. Firstly, the mode volume can be very small (e.g. in the subwavelength scale). Secondly, the resonator is open (no reflecting walls are utilized) and resonant effects can be found in a continuous spectrum (i.e. a continuum of eigenmodes). Thirdly, we only need one homogenous medium to realize the proposed resonator. The shape of the resonator can be a ring structure of arbitrary shape. In addition to the natural applications (e.g. optical storage) of an optical resonator, we also suggest some other applications of our novel optical open resonator (e.g. power combination, squeezing electromagnetic energy in the free space).

Cavities/resonators have been widely utilized to confine electromagnetic energy^1^,^2^. The mode volume and the quality factor *Q* are two important properties of an optical cavity/resonator, reflecting its ability to confine the light in the spatial domain and the time domain, respectively. Surface plasmon polariton cavities can confine the light in a subwavelength scale^3^. However, the quality factor *Q* is limited due to the loss in the metal. Dielectric cavities can achieve a high *Q*^4^, although the whole structure (e.g. a photonic crystal cavity) is usually much larger than the operating wavelength. One approach for compromise is to combine the metals and dielectric materials together to form a composite cavity, similarly to designing a hybrid dielectric-plasmonic waveguide that can confine the light on a subwavelength scale with low loss^5^.

Transformation optics (TO) is a powerful theoretical tool that can be utilized to design many novel optical/electromagnetic devices with pre-designed functions^6^,^7^,^8^. V. Ginis’s group has used TO to design many novel optical cavities that can confine the light on a subwavelength scale with an extremely high *Q* factor^9^,^10^. Many different types of coordinate transformations have been given in their studies, and the theory on how to design an optical cavity/resonator with an unlimited quality factor and subwavelength mode volume by the coordinate transformations is available. There are also some other studies on designing an optical cavity by TO^11^,^12^. These cavities designed by TO are open cavities, which can confine the light wave inside without any reflecting walls. For the traditional cavity/resonator (e.g. with some reflecting walls), a standing wave is formed inside the cavity/resonator at the resonance frequency. For an open cavity/resonator, the light path is cancelled inside the cavity. We should also note that in addition to using TO, open cavities can also be designed by using alternating positive and negative refraction index media^13^,^14^.

The basic idea of a novel method for designing optical devices, namely the optical surface transformation (OST), is proposed in a short report^15^, and a detailed theory has been given later^16^. In this paper we use the OST to design a novel zero-order optical resonator that has the following features. Firstly, the size of the resonator can be either very large or very small compared to the working wavelength (i.e. a subwavelength mode volume can be achieved). Secondly, the resonant frequencies can be a continuous spectrum in theory. Note that in practice the resonant frequencies will be influenced by the dispersion properties of the real materials used (e.g. metamaterials or photonic crystals). Thirdly, the proposed resonator is an open resonator, which does not contain any reflecting walls. The resonant effect is due to the cancellation of the optical path like in other open cavities^14^.

Our FDTD simulations show that the *Q* factor of the proposed cavity is sensitive to the loss of a zero refractive index metamaterial with the Drude model (e.g. the substantial loss in the Drude model will greatly influence the performance of the proposed cavity). We will give the detailed analysis about the loss effect to our cavity and the methods to tackle this problem in the discussion part.

## Materials and Methods

### The Optical surface transformation and the optic-null medium

The optical surface transformation (OST) has been recently proposed^16^. Here we just use the final conclusion given in Ref. 16 to design the novel zero-order optical resonator. As shown in Ref. 16, two arbitrarily shaped surfaces connected by the optic-null medium (ONM) can perform equivalently (i.e. they correspond to the same surface in the reference space, and the wave propagates from one surface to the other without any phase delay). An ONM is a highly anisotropic medium whose relative permittivity and permeability are extremely large along its main axis and nearly zero in other orthogonal directions. Such an ONM has been experimentally demonstrated by metamaterials^17^,^18^. For example, the ONM with the main axis in the *x* direction can be expressed by: *ε*_*x*_ = *μ*_*x*_ = 1/Δ, *ε*_*y*_ = *μ*_*y*_ = *ε*_*z*_ = *μ*_*z*_ = Δ, Δ → 0. The ONM with the main axis in the radial direction can be given by: *ε*_*r*_ = *μ*_*r*_ = 1/Δ, *ε*_*θ*_ = *μ*_*θ*_ = *ε*_*z*_ = *μ*_*z*_ = Δ, Δ → 0.

**Figure 1:**
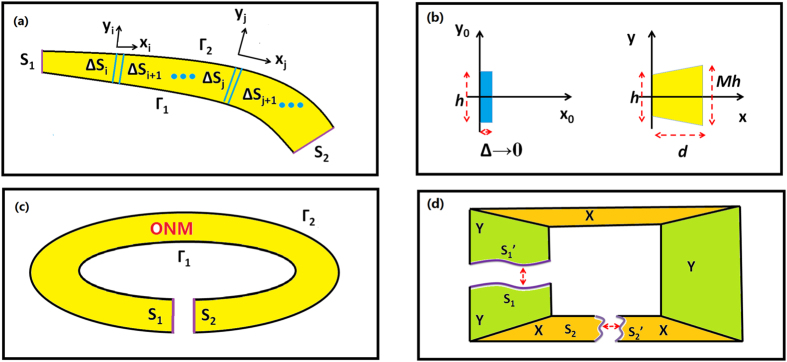
(**a**) Two arbitrarily shaped surfaces *S*_1_ and *S*_2_ as the terminated surfaces of two conformal smooth curves *Γ*_1_ and *Γ*_2_ (in accordance with the normal direction of these two smooth curves) will perform equivalently, if the ONM given in Eq. (2) is filled inside the each sub-region between *Γ*_1_ and *Γ*_2_ (the yellow region). (**b**) The coordinate transformation relation in each small sub-region. The left is the reference space (*x*_0_, *y*_0_, *z*_0_), and the right is the real space (*x*, *y*, *z*) which corresponds to the local coordinate (*x*_i_, *y*_i_, *z*_i_) in (**a**). When Δ → 0, the blue volume in the reference space reduces to a surface which corresponds to the yellow region (i.e. the ONM) in the real space. If there is some scaling along the *y* direction (i.e. *M* ≠ 0), the main axis of the ONM is not along the *x* direction (see Eq. (2)). If there is no compression and extension along *y* direction (i.e. the normal direction of the conformal curves in (**a**)), we have *M* = 1, which means the main axis of the ONM is along the *x* direction (i.e. the tangential direction of the conformal curves in (**a**) and see Eq. (3)). (**c**) A closed loop is formed if we connect *S*_1_ and *S*_2_ together. (**d**) another way to design an optical resonator by the OST: *S*_1_ and *S*_2_ have been linked by the ONM. *S*_1_’ and *S*_2_’ have exactly the same shape as S_1_ and S_2_, respectively. *S*_1_’ and *S*_2_’ are also linked by the ONM. If we connect *S*_1_ and *S*_1_’, *S*_2_ and *S*_2_’ together, respectively, a closed loop filled with the ONM has been created. Such closed loop performs like an optical open resonator. The orange and green regions labeled by ‘X’ and ‘Y’ stand for the ONM with main axis along the *x* and *y* directions, respectively.

We first show that two arbitrarily shaped surfaces linked by the ONM enclosed by two smooth curves also perform equivalently (see Fig. 1(a)) and then conclude that if the two curves are conformal (i.e. the tangential directions of the two conformal smooth curves should be the same at each part), the main axis of the ONM inside the two conformal smooth curves is the same as the tangential direction of the curves. This can be proved by dividing the whole ONM into many small regions along the normal direction of one smooth curve *Γ*_1_ (see Fig. 1 (a)). We can set up a local Cartesian coordinate system in each small region (the local *x* coordinate variable is along the tangential direction of one curve *Γ*_1_, and the local *y* coordinate variable is along the normal direction). In each small region, two surface elements (e.g. Δ*S*_i_ and Δ*S*_i+1_, or Δ*S*_j_ and Δ*S*_j+1_), linked by the ONM with the main axis along the local *x* direction, perform equivalently. This relationship will transfer from *S*_1_ to *S*_2_, and hence surfaces *S*_1_ and *S*_2_ perform equivalently (i.e. any point source on surface *S*_1_ will produce a corresponding image on surface *S*_2_, and the light wave propagating from *S*_1_ to *S*_2_ will not produce any phase delay). In each small region, the coordinate transformation between the reference space and the real space can be given as (see Fig. 1(b)):


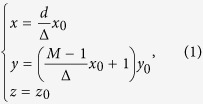


where (*x*, *y*, *z*) and (*x*_0_, *y*_0_, *z*_0_) denote the coordinates in the real and reference space, respectively, and *M* determines the compression factor along the *y* direction. The relative permittivity and permeability in this small region can be calculated with the help of TO:


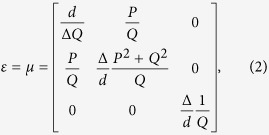


where 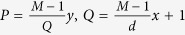. When Δ → 0, the medium will reduce to the ONM. However if *M* ≠ 1 (i.e., there is some scaling along the normal direction of two conformal curves, which means that the two curves are not conformal), the main axis of the ONM described by Eq. (2) will not be along the local *x* direction (i.e. the tangential direction of curve *Γ*_1_). If we assume the tangential directions of the two curves (i.e. *Γ*_1_ and *Γ*_2_) are the same in each small region, which also means that the area of the cross section between two curves *Γ*_1_ and *Γ*_2_ is a constant in our design (i.e. *M* = 1 in all small regions), then Eq. (2) reduces to


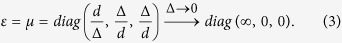


Equation (3) shows that the medium in each small region (e.g. region between Δ*S*_i_ and Δ*S*_i+1_ in Fig. 1(a)) is always the ONM with main axis along the local *x* direction (i.e. the tangential direction of the two conformal curves) if two curves (i.e. *Γ*_1_ and *Γ*_2_) are conformal (i.e. the tangential directions of the two curves are the same in each small region).

### Achieving an optical resonator by the optic-null medium

Consider one special case in which the shape of the two surfaces *S*_1_ and *S*_2_ are the same, and imagine that the ONM connected to the two arbitrarily shaped surfaces performs like a cable that can be bent smoothly. If we connect *S*_1_ and *S*_2_ together, we will obtain a closed loop filled with the ONM (see Fig. 1(c)), which will perform like a novel optical resonator. The shape of such a loop can be arbitrary (provided that the inner and outer boundaries of this loop are conformal smooth curves).

First we introduce a simple way to design an open resonator by OST. We begin by choosing the shape of a closed loop whose inner and outer boundaries are conformal smooth curves (i.e., the two curves always have the same local tangential direction). The medium inside the loop is the ONM whose main axis is along the tangential direction of the two conformal curves. Such a closed loop filled by the ONM performs like a special optical open resonator. A circular ring structure is the simplest shape.

**Figure 2:**
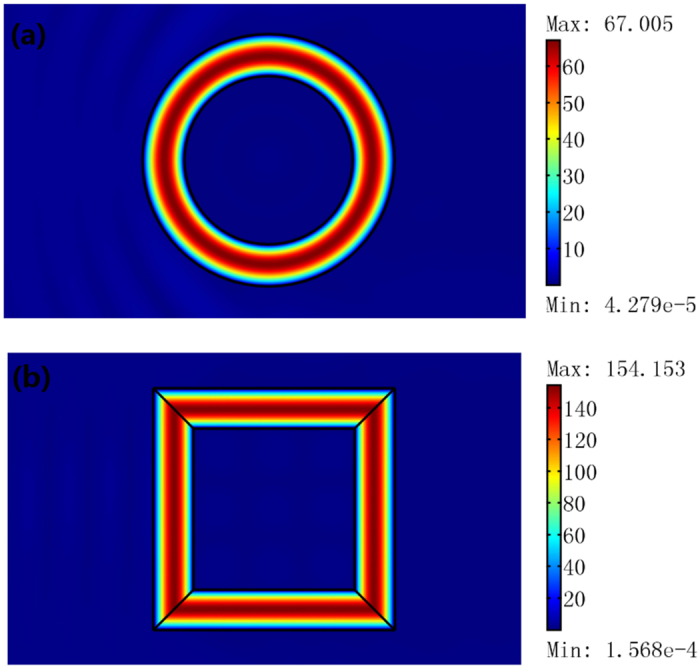
The 2D FEM simulation results for a TE wave case. A plane wave with unit amplitude is incident onto the resonator from the left. We plot the absolute value of the electric field’s *z* component. (**a**) A concentric ring resonator with inner radius λ_0_ and outer radius 1.5λ_0_. (**b**) A rectangular ring resonator with inner side length of 2λ_0_ and outer side length of 3λ_0_.

The way to design an open resonator by the OST is not limited to the above simple way. Actually the OST can make two arbitrarily shaped surfaces *S*_1_ and *S*_2_ equivalent (see Fig. 1(d)). We can also design some ONM to make equivalent another pair of two surface *S*_1_’ and *S*_2_’, which have exactly the same as shapes as *S*_1_ and *S*_2_, respectively. If we connect *S*_1_ and *S*_1_’, *S*_2_ and *S*_2_’ together, respectively, we will obtain a closed loop filled with ONM, performing like an open resonator (see Fig. 1(d)). *Γ*_1_ and *Γ*_2_ are not necessarily smooth conformal curves (i.e. they can have some sharp corners where we cannot define the tangential direction). An example designed by this method is shown in Fig. 2(b).

## Results and Discussion

### The performance of the optical resonator

**Figure 3:**
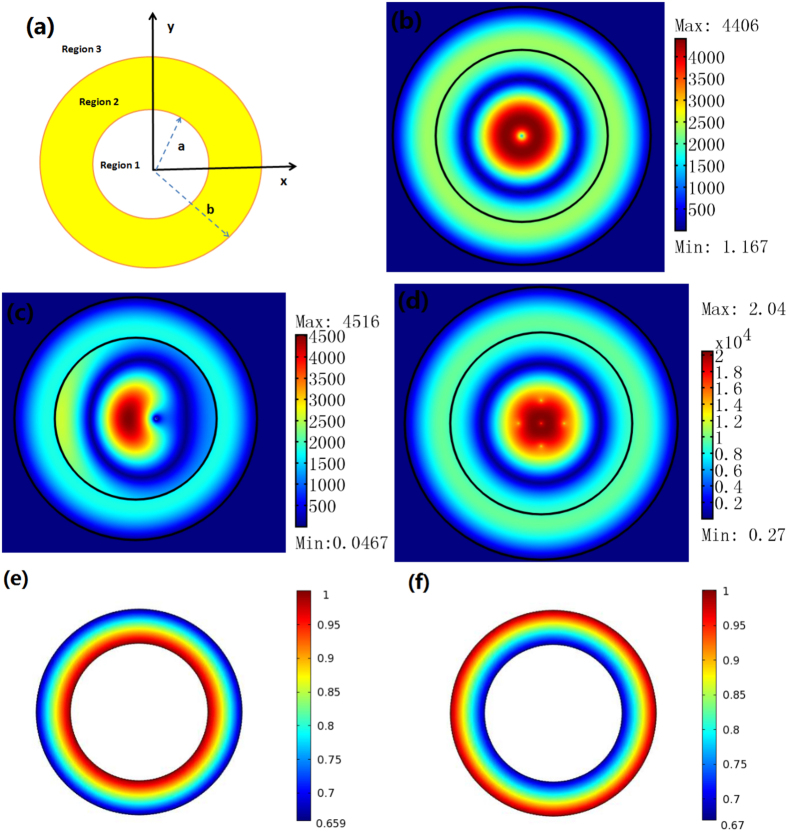
(**a**) A 2D open resonator structure we study here: regions 1 and 3 are free space. Region 2 is the concentric ring open resonator filled with the ONM whose main axis is in the *θ* direction. (**b**–**d**) are 2D FEM simulation results for a TE wave case. We plot the absolute value of the electric field’s *z* component. (**b**,**c**) we set a line current with amplitude 1A in the center and deviated λ_0_/6 from the center of Region 1, respectively. (**d**) We set five line currents with amplitude 1A in Region 1. The size of the resonator is the same from (**b**–**d**): *a* = 2λ_0_/3, *b* = λ_0_. (**e**,**f**) are two normalized eigenmodes of the resonator with the same size as (**b**) (obtained by the FEM numerical method), which correspond to the zero-order Bessel function and the zero-order Neumann function in Eq. (12), respectively.

We use the finite element method (FEM) to simulate the performance of the proposed resonator. For simplicity, we consider a 2D concentric ring resonator (i.e. the inner and the outer boundaries of the cavity are both circles, the main axis of the ONM filled in the cavity is in the *θ* direction). The structure of this open resonator is given in Fig. 3(a). If we set a unit line current at the center of the concentric ring, the mode in the 2D concentric ring resonator is excited (see Fig. 3(b)). We should note that there is no resonant frequency for this resonator (i.e. if we change the frequency of the line current, the mode in the open resonator can still be excited). We can also analyze the eigenmode of the resonator and explain this phenomenon analytically due to the simple geometry we choose here. The Helmholtz equation for a 2D TE wave propagation in an anisotropic medium in a cylindrical coordinate system can be given by:


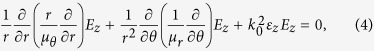


where *μ*_r_ and *μ*_θ_ are the relative permeabilities in the radial and tangential directions, respectively, and *ε*_z_ is the relative permittivity in the *z* direction. The ONM is a homogenous medium, and hence we can rewrite Eq. (4) as:


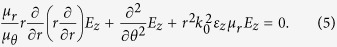


We can obtain the general solution of Eq. (5) by separation of variables:


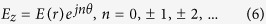


*E*(*r*) satisfies the following Bessel equation:


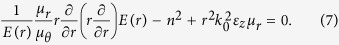


Considering the medium of an ONM in this 2D concentric ring structure:


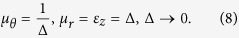


We can rewrite Eq. (7) as:


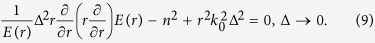


In order to have a non-zero solution *E*(*r*) in Eq. (9), it requires *n* = 0, and hence Eqs (6) and (9) can be reduced to


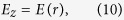



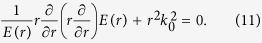


Equation (11) is a zero-order Bessel equation, and hence the solution to Eq. (4) in a concentric ring ONM can be written as:


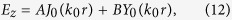


where *A* and *B* are constant, which can be fixed by the boundary condition that is related to the method of excitation. The eigenmode of this 2D concentric ring resonator is the linear superposition of the zero-order Bessel function and the zero-order Neumann function. That is the reason why we call it as the zero-order resonator. The resonance frequency is a continuous spectrum (no reflecting wall is utilized in this open resonator and the natural periodic boundary condition in the *θ* direction is removed by the ONM).

Next we will study the case when we set a unit line current in the center of this 2D concentric ring resonator. Then the electric field’s *z* component in each region (defined in Fig. 3(a)) can be written as:


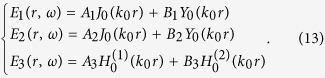


Note that the field in Region 2 is the eigenmode of the concentric ring ONM given in Eq. (12). The field in Region 1 contains a singularity as a line current source is set in the center (*B*_1_ ≠ 0). The field in Region 3 should be an outgoing cylindrical wave (no energy comes from beyond), and hence *B*_3_ = 0 (the time harmonic is chosen as exp(−i*ωt*)).

Considering the boundary condition that the electric field’s tangential component should be continuous at the boundary, we can obtain:


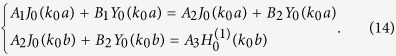


where *a* and *b* are the inner and outer radii of the concentric ring, respectively (see Fig. 3(a)). The magnetic field’s tangential component can be determined from the electric field’s *z* component:


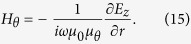


By combining Eqs (8), (13) and (15), we can obtain (*B*_3_ = 0):


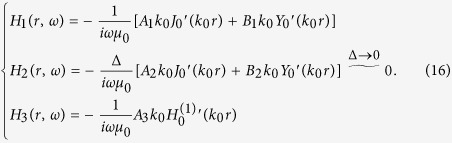


Considering that the magnetic field’s tangential component should also be continuous at the boundary, we can obtain:


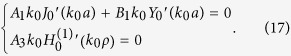


By combining Eqs (14) and (17), we can obtain the coefficients in Eq. (13):


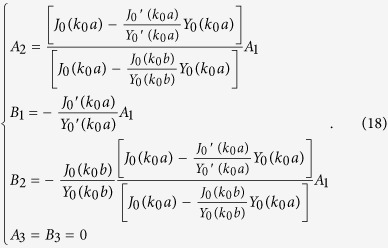


One important feature from Eq. (18) is that the field in Region 3 is zero, which is consistent with the FEM simulation (see Fig. 3(b)). Note that in numerical simulation, we use an extremely large number and an extremely small number (e.g. 1000 and 0.001) to approximately simulate the ONM (but not ideally infinity and zero). Thus, a very small electric field enters Region 3 in the simulation.

**Figure 4:**
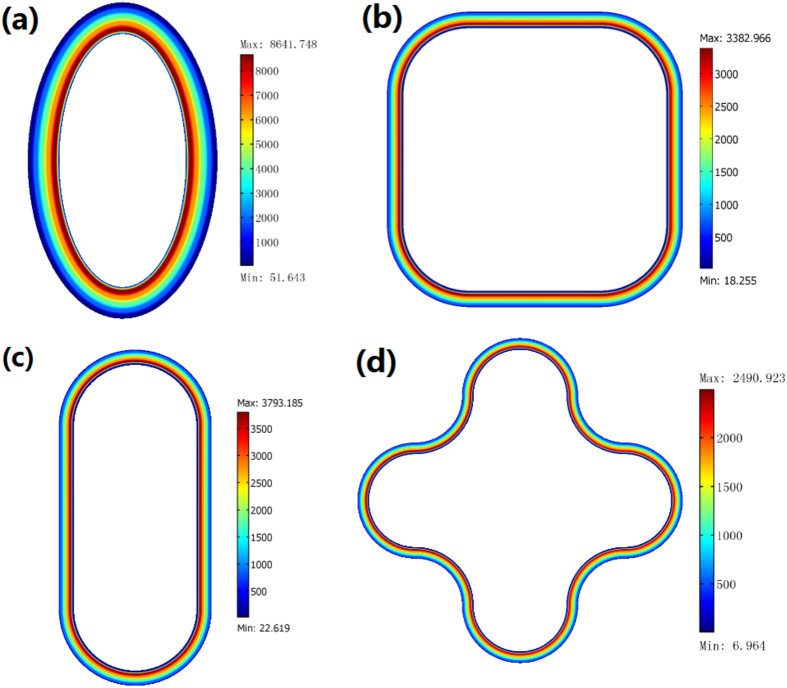
Some cavities of other shapes designed by the OST. We only plot the absolute value of electric field’s *z* component inside the resonator for the TE polarization. We use one line current source with amplitude 1A inside the loop to excite the mode. (**a**) an elliptic resonator. (**b**) a rectangular resonator with smooth corners. (**c**) a playground shaped resonator. (**d**) a petaloid resonator. The whole size of these resonators are all smaller than the wavelength in above simulations.

Note that to excite the eigenmode the line current is not necessarily at the center of the concentric ring: a deviated line current (see Fig. 3(c,d)) or a plane wave (see Fig. 2(a)) can also excite the eigenmode of the resonator. The eigenmodes of the resonator are calculated with the FEM and shown in Fig. 3(e,f), which corresponds to the zero-order Bessel function and the zero-order Neumann function in Eq. (12). The shape of the open resonator is not necessarily a concentric ring structure and can be designed in some other structures (e.g. in Fig. 4). In practice, we can choose an open resonator of an appropriate shape to get high efficiency of excitation (e.g. a concentric ring cavity for a line current excitation, and a rectangular ring resonator for a plane wave excitation).

### The applications of the novel optical zero-order resonator

In addition to the traditional applications of an optical resonator (e.g. to confine electromagnetic energy), our open optical resonator designed with OST has many other novel applications. Here we list three important applications of the open resonator proposed in this paper. Firstly it can be utilized as an electromagnetic energy collector. As shown in Fig. 3(d), we set five line currents in Region 1 outside the concentric ring resonator, and obtain a higher field inside the resonator (compared with a single line current case in Fig. 3(b,c)).

**Figure 5:**
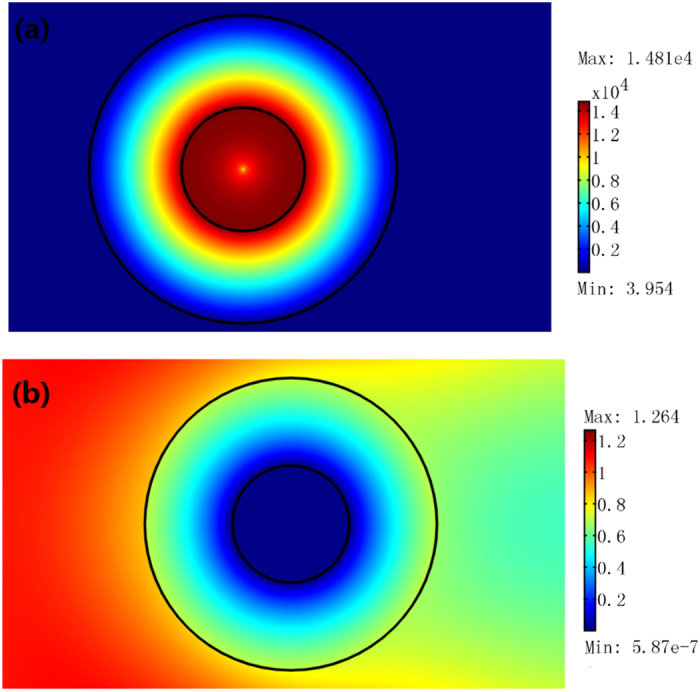
The 2D FEM simulation results for a TE wave case. We plot the absolute value of the electric field’s *z* component. The open concentric ring resonator here has a subwavelength size (e.g. *a* = λ_0_/15, *b* = λ_0_/6). (**a**) A line current with amplitude 1A is set at the center of the concentric ring resonator. (**b**) A plane wave with unit amplitude is incident onto the resonator from the left.

Secondly, the resonator composed by the ONM can be of a subwavelength size (see Fig. 5(a,b)), allowing it to function as an optical open micro-resonator for concentrating the light at the subwavelength scale.

**Figure 6:**
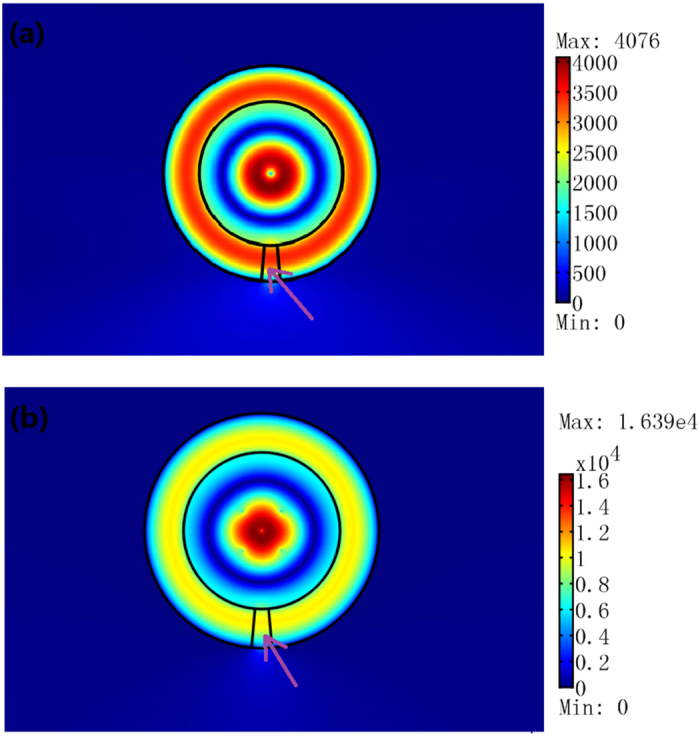
The 2D FEM simulation results for a TE wave case. We plot the absolute value of the electric field’s *z* component. We cut off a small region from the concentric ring resonator in Fig. 3(a). The inner and outer radii of the ring are *a* = 2λ_0_/3 and *b* = λ_0_, respectively. (**a**) We set one line current with unit amplitude in the center of the ring. (**b**) We set five line currents with unit amplitude in the central air region of the ring resonator. The electric field is enhanced in the air gap region of the ring resonator. The pink arrow indicates the location of the air gap in the resonator.

Thirdly, we can achieve an electric field enhancement in a region of air after some small modifications of our open resonator. For example, we can cut off a small region from the concentric ring resonator in Fig. 3(a) (i.e. the ring with a small air gap). As shown in Fig. 6(a), we can obtain an enhanced electric field in the air gap region of the ring resonator. Furthermore, we can acquire a higher field in the air gap region simply by adding more sources in the central air region of the ring resonator (see Fig. 6(b)).

### Comparison with other cavities designed by TO

Optical open cavities/resonators composed of a complementary medium can be explained by TO: it can be treated as the folding of the space repeatedly along the *θ* direction. The effective optical path is zero (i.e. the space is folded to null), and there is no limitation on the resonance frequencies^13^,^14^.

Some novel cavities/resonators have also been designed by TO^9^,^10^,^11^,^12^. All these cavities/resonators have a continuous resonance spectrum and do not need any reflecting walls. The reason for this can be easily understood: all these cavities/resonators are transformed from a free reference space to the real space (i.e. there is no cavity with reflecting walls in the reference space). This means that the resonance frequency is a continuous spectrum in the reference space. The coordinate transformation simply changes the field distribution but does not influence the resonance frequencies, and hence all these cavities/resonators have a continuous resonance spectrum. As an open resonator proposed in this paper, we connect two equivalent surfaces linked by the ONM together to form a closed loop filled with the ONM whose main axis is along the tangential direction of the two conformal boundaries of the loop. All the surfaces perpendicular to the main axis of the ONM inside the resonator are equivalent surfaces (e.g. surfaces perpendicular to the *θ* direction of a circular ring resonator), and hence when the light propagates along the tangential direction of the resonator (i.e. the direction of the ONM’s main axis), there is no phase delay (i.e. the effective light path is zero). Note that The physical mechanism of the open resonator composed by ONM is similar to the open cavity composed by positive and negative refraction materials (i.e. the cancellation of the light path)^13^,^14^. Actually a pair of positive and negative refraction materials perform equivalently like an ONM^19^.

### The physical mechanism of the open resonator

To further understand the physical mechanism of the open resonator composed by ONM, we make the following simulations of wave guiding (along the closed loop filled with the ONM) for 2D TE polarization case (i.e., the electric field is along the *z* direction while the magnetic field is in the *x*−*y* plane; consequently the light path should be determined by *n*_*r*_ and *n*_*θ*_, besides the propagation length:


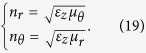


**Figure 7:**
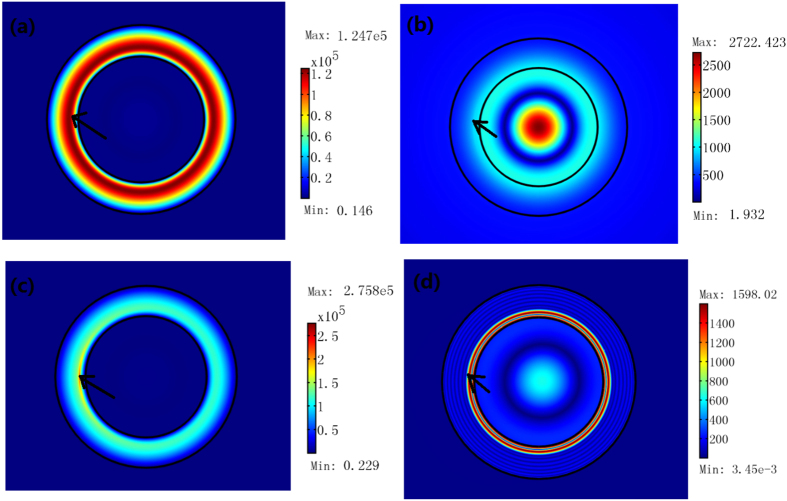
The 2D FEM simulation results for a TE wave case. We plot the absolute value of the electric field’s *z* component. We use one line current source with amplitude 1A inside the loop (indicated by the black arrow) to excite the mode. (**a**) The open circular resonator is composed by the ONM with *μ*_θ_ = 10000 and *μ*_r_ = *ε*_z_ = 0.0001. (**b**) *μ*_θ_ = 1 and *μ*_r_ = *ε*_z_ = 0.0001. (**c**) *μ*_θ_ = 10000, *μ*_r_ = 1, and *ε*_z_ = 0.0001. (**d**) *μ*_θ_ = 10000, *μ*_r_ = 0.0001, and *ε*_z_ = 1. The size of the cavity is chosen as *a* = 2λ_0_/3, *b* = λ_0_. We also use FDTD simulation to calculate the *Q* factor for each case. In the FDTD simulation, we assume the dispersion relation in the simulation to satisfy the required material parameters at the resonance frequency λ_0_. *Q* factor in (**a**,**c**) are both very large around the resonant frequency. Q factors in (**a**,**c**) are very large (over million) at the resonant frequencies, while no resonance mode appears for (**b**,**d**) in the FDTD simulation.

For an ONM, the effective refraction index is 1 and zero along *r* and *θ* direction, respectively (e.g. *μ*_θ_ = 1/Δ, and *μ*_r_ = *ε*_*z*_ = Δ, where Δ → 0). If we replace *μ*_θ_ by air while keeping other parameters unchanged (e.g. *μ*_r_ and *ε*_z_ are both chosen as nearly zero), the effective refraction index of the ring resonator along *r* direction decreases, and hence some energy inside the resonator will be leaked out into the surrounding air region (see Fig. 7(b)). In this case, there is no resonance mode in a FDTD simulation (i.e. *Q*-factor drops to zero in Fig. 7(b)).

If we replace *μ*_r_ by air, while keeping other parameters unchanged, the effective refraction index of the ring resonator along *θ* direction increases (see Eq. (19)). In this case, and the model pattern changes (see Fig. 7(a,c)). Note that the resonance frequency and the *Q*-factor also slightly changes in the FDTD simulations. However we can still find a resonant mode in this case. In the FDTD simulation, the material dispersion is considered, and hence the resonant frequency is no longer continuous.

If we replace *ε*_z_ by air, while keeping other parameters unchanged, both n_*r*_ and n_*θ*_ increase. There is also some energy leakages from the resonator (see Fig. 7(d)). In this case, the resonance mode also disappears in the FDTD simulation.

Now we can summarize different roles of *n*_r_ and *n*_θ_ from above analyses: *n*_r_ → 1 can prevent the energy leakage from the ring resonator or attract the wave from the surrounding space. *n*_θ_ → 0 can ensure the effective light path is zero along the *θ* direction and provide a high *Q*-value of the resonator.

**Figure 8:**
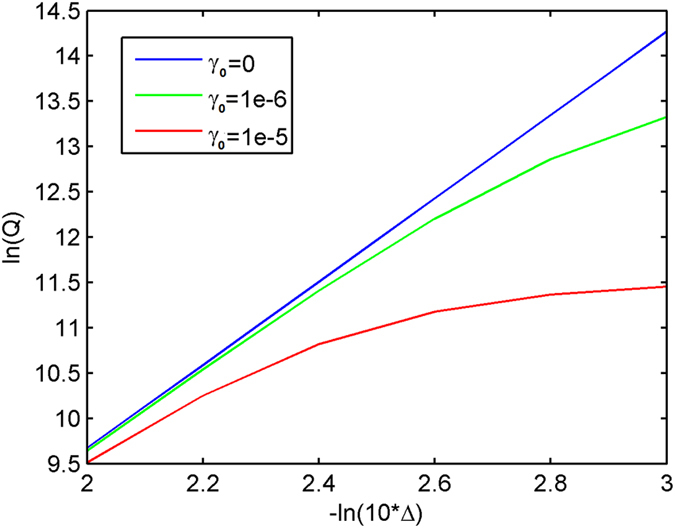
The FDTD simulation results: the relation between Δ and the *Q* factor of the concentric ring resonator with size *a* = λ_0_ and *b* = 1.5λ_0_. λ_0_ is the resonance wavelength. Here the permittivity and permeability of the cavity is given by the Drude model of Eq. (20) with normalized plasma frequency *ω*_p_. If *γ*_0_ = 0 and Δ → 0 (e.g. –ln(10*Δ) approaches infinity), the *Q* factor approaches infinity. Different colored lines are for different values of the damping coefficient *γ*_0_. For a fixed small Δ, the *Q* factor drops as the damping coefficient increases, as expected.

In a practical application, the ONM can be approximately realized by the medium whose relative permittivity and permeability are far larger than 1 in the main axis direction (not necessary infinitely large) and between 0 and 1 (not necessarily zero) in other orthogonal directions, which corresponds to the case that Δ in Eq. (3) is not exactly zero. We also study the *Q* factor of the proposed resonator when Δ changes. As shown in Fig. 8, the *Q* factor increases as Δ approaches zero (the *Q* is infinite if Δ = 0 for a lossless resonator in theory).

**Figure 9:**
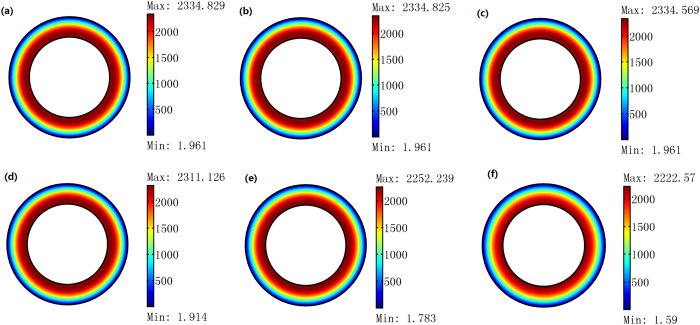
The 2D FEM simulation results for a TE wave case. We plot the absolute value of the *z* component of the electric field inside the circular cavity when the loss tangent *δ* changes. *δ* = 0, 0.001, 0.01, 0.1, 0.2, 0.3 [rad] from (**a**–**f**). The size of the cavity and the excitation method are the same as those for Fig. 3(b).

To realize the proposed optical cavity, we may need some metamaterial with nearly zero permittivity and permeability, which necessarily has substantial loss^20^. We study the loss effect on the performance of the proposed cavity. First we use the FEM simulation to see whether the loss will influence the mode pattern of the cavity (see Fig. 9). We introduce the loss tangent that is defined by tan *δ* = Re{*ε*}/Im{*ε*} = Re{*μ*}/Im{*μ*} as a varying parameter in our simulations. As shown in Fig. 9, the mode pattern of the cavity is insensitive to the loss.

Through FDTD simulations we also study the performance of the proposed optical cavity when the loss consistent with Kramer-Kronig relations (i.e. the causality) is considered. We assume the material’s permittivity and permeability satisfy the Drude model (i.e. the Kramer-Kronig relation is naturally satisfied):^21^


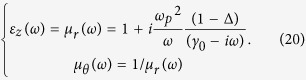


The relation between *Q* factor and Δ is given in Fig. 8 for several different values of damping coefficient (i.e. γ_0_ in Eq. (20)). For a fixed small Δ, the *Q* factor drops as the damping coefficient increases, as expected. When the damping coefficient *γ* reaches a relatively large number (e.g. 0.2 or more), we cannot find any resonance peak any more in the FDTD simulation, due to the resonance spectrum broadening caused by the loss and resonance peak shift caused by the increased real part of the permittivity (see Eq. (20)). It reveals the fact that a substantial loss in the Drude model will greatly influence the performance of the proposed cavity.

One possible way to keep a large *Q* factor in a future experiment is to use all-dielectric photonic crystal structure to realize the zero refractive index at the Dirac point frequency, which has been experimentally demonstrated in both microwave^22^ and optical frequencies^23^. Low loss zero refractive index materials can also be realized by introducing some gain media^24^,^25^.

Another way to keep the performance of the proposed cavity is to use some other equivalent/effective metamaterials (without any zero refractive index material component) to realize the proposed cavity composed by the ONM. Actually the optic-null medium (ONM) has been experimentally demonstrated by many different metamaterials without any zero refraction index material component^17^,^18^,^26^. For example, a metallic plate with fractal holes has been theoretically shown to perform like an ONM and experimentally realized for a subwavelength imaging^17^. A metallic slit array satisfying the Fabry-Pérot (FP) resonances condition can also perform like an ONM, which has also been experimentally demonstrated for an electromagnetic wave concentrator at a microwave frequency^18^. These experimental results show that the loss in real metal does not influence the performance of the ONM, and may provide a more practical way to realize the proposed cavity by the OST and ONM.

The zero-order optical resonator designed by an OST in this paper has some other special features. Firstly the method to design such a resonator is very simple: what we need to do is just design two conformal smooth closed curves according to the occasion of application (e.g. the method of the excitation), and fill an ONM inside this ring structure. Secondly, we only need one homogeneous medium (i.e. the ONM) to realize the resonator designed by the OST (e.g. without any gradient control), which is a significant advantage compared with other cavities/resonators design by TO. Thirdly, the resonator proposed here can be an optical open micro-resonator that confines the electromagnetic energy on a subwavelength scale. The proposed resonator can also perform as an electromagnetic energy collector or a power combination device (see Fig. 3(d)). We can also achieve an electromagnetic energy squeezing effect in a region of air by cutting an air gap from the ring resonator (see Fig. 6).

## Conclusions

A novel optical zero-order resonator has been designed by OST in this paper. Compared with the optical cavities/resonators designed by TO^9^,^10^,^11^,^12^, the optical resonator designed by OST here have many other special features. Firstly, we do not need any detailed coordinate transformation. The designing process with an OST is very simple: we just need to choose the shape of the resonator, and the medium inside the resonator will be naturally determined, as we have explained in the method section. Secondly, the shape of our resonator can be an arbitrarily shaped ring structure (not necessary a circular ring structure). We can choose resonators of different shape according to different forms of excitation. Thirdly, we only need one homogeneous anisotropic medium without any left-hand materials to realize the open resonator proposed in this paper. The optic-null medium (ONM), which has been experimentally realized in microwave frequencies^17^,^18^, is such a medium. The proposed resonator will have many potential and novel applications (e.g. capturing electromagnetic waves, collecting electromagnetic energy, squeezing electromagnetic energy in a region of air, etc.) in the future.

## Additional Information

**How to cite this article**: Sun, F. *et al.* Creating a zero-order resonator using an optical surface transformation. *Sci. Rep.* **6**, 21333; doi: 10.1038/srep21333 (2016).
